# miR-320b suppresses cell proliferation by targeting c-Myc in human colorectal cancer cells

**DOI:** 10.1186/s12885-015-1728-5

**Published:** 2015-10-20

**Authors:** Hantao Wang, Fuao Cao, Xu Li, Hua Miao, Jifu E, Junjie Xing, Chuan-gang Fu

**Affiliations:** 1 Department of Colorectal Surgery, Changhai Hospital, Shanghai, 200433 China; 2 Department of General Surgery, The First People’s Hospital of Pinghu, Pinghu, 314200 Zhejiang Province China

**Keywords:** Colorectal cancer (CRC), miR-320b, c-Myc, Cyclin D1

## Abstract

**Background:**

MicroRNAs (miRNAs) are small noncoding RNAs that potentially play a critical role in tumorigenesis. Mounting evidence indicates that one specific miRNA: miR-320b is down regulated in numerous human cancers, including colorectal cancer (CRC); making the hypothesis that miR-320b may play a key role in tumorigenesis plausible. However, its role in carcinogenesis remains poorly defined. The goal of this study is to better clarify the role of miR-320b in tumor growth of CRC.

**Methods:**

Quantitative reverse-transcription polymerase chain reaction (qRT-PCR) was conducted to detect the expression of miR-320b in CRC tissues and 5 CRC cell lines. The effect of miR-320b on cell proliferation was analyzed *in vitro* and *in vivo*. Furthermore, a luciferase reporter assay was performed to measure the target effects of miR-320b. Lastly, the messenger RNA (mRNA) and protein levels of the gene *c-MYC* were measured in CRC cell lines and tissues by qRT-PCR, and confirmed via Western blot and Immunohistochemical (IHC) staining.

**Results:**

The results presented here showed that miR-320b expression was down regulated in both CRC tissues and cells. Overexpression of miR-320b in CRC cells was statistically correlated with a decrease of cell growth *in vitro* and *in vivo*, while *c-MYC* was identified as a target gene of miR-320b in CRC. Furthermore, it was found that up-regulation of c-Myc can attenuate the effects induced by miR-320b.

**Conclusions:**

Our identification of *c-MYC* as a target gene of miR-320b provides new insights into the pathophysiology of CRC proliferation, and identifies miR-320b as a novel therapeutic target for the treatment of CRC.

## Background

Colorectal cancer (CRC) is one of the most common cancers, and accounts for approximately 9.4 % of all cancer cases worldwide. According to the International Agency for Research on Cancer (IARC), approximately 1 million new cases are detected each year [1, 2]. There has been considerable effort devoted to investigating the biological mechanisms responsible for promotion of CRC; however, the detailed mechanisms underlying CRC pathogenesis remain elusive.

MicroRNAs (miRNAs) are a class of small RNAs (~22 nucleotides) down regulates the expression of their target genes through mRNA degradation or translational inhibition [3, 4]. The target site of miRNA has been considered to be the 3′ un-translated region (3′ UTR) of mRNA. However, increasing evidence shows that miRNAs may also bind the coding regions or the 5′ un-translated region (5′ UTRs) [5, 6]. Mounting evidence indicates that post-translational regulation of gene expression mediated by miRNAs acts as either a tumor suppressor or an oncogene in CRC [7–11]. Several miRNAs found in CRC such as miR-451, miR-34, miR-135, miR-122, let-7a, miR-143, miR-17-92, miR-101, miR-126, miR-200, miR-203, miR-183 and miR-21 have been shown to have an effect on cell growth and apoptosis; as well as invasion and migration [11]. Previous studies have shown that miR-320b is significantly down regulated in several cancers including CRC [12], glioblastoma [13], gastric cancer [14] and bladder cancer [15]. Given that down regulation of miR-320b is common in a number of cancers, it has been hypothesized that miR-320b may play an important role in tumor development and tumorigenesis.

In this study, we evaluated the miR-320b expression levels in tumor tissues from 48 CRC patients and found that miR-320b was significantly down regulated in these tissues. Additionally, it was found that the overexpression of miR-320b in CRC cells was correlated with observed decreases in cell growth both *in vitro* and *in vivo*. This study also demonstrated that *c-MYC* was directly targeted by miR-320b, and that overexpression of miR-320b in CRC cells decreased both mRNA production, and protein expression of *c-MYC*. These findings strongly suggested that miR-320b inhibited proliferation of CRC cells through an inhibition of *c-MYC* gene function, and that increasing miR-320b expression levels may provide a novel approach for CRC treatments.

## Methods

### Tissue samples and cell lines

A total of 48 CRC tissue samples and their adjacent non-tumor tissues were obtained from Department of Colorectal Surgery, Changhai Hospital (Shanghai, China) for qRT-PCR analysis. All tissue samples were obtained surgically and immediately snap frozen and stored in liquid nitrogen until use. The study protocol was approved by Shanghai Changhai Hospital Ethical Committee, and written informed consent was obtained from all subjects before the study was conducted. Additionally, five normal colorectal tissues were obtained from non-cancer patients by colonoscopy. For the *in vitro* experiments, cell lines including HCT-116, SW-480, SW-620, LoVo and HEK293 were used and were purchased from American Type Culture Collection (ATCC). SW-480 and SW-620 were cultured in Leibovitz’s L-15 medium containing 10 % FBS. HCT-116 and LoVo cells were cultured in Ham’s F12K medium containing 10 % FBS, and HEK293 cells were cultured in DMEM medium containing 10 % FBS. All cells were maintained at 37 °C in a humidified atmosphere with 5 % CO_2_.

### RNA quantification

Total RNA was isolated using a Trizol extraction kit (Life Technologies, USA) according to the manufacturer’s instructions. Purified mRNA and miRNAs were detected by qRT-PCR assay using All-in-One miRNA qRT-PCR Detection Kit (GeneCopoeia, USA). U6 small RNA was used as an internal control for normalization and quantification of miR-320b expression. As an internal control β-actin was measured for normalization and quantification of c-Myc expression.

### Luciferase reporter assay

The luciferase reporter was constructed by cloning human *c-MYC* cDNA sequence into pMIR-Report (Ambion, Austin, USA). Wild type or mutant *c-MYC* mRNA fragments were amplified and cloned into the luciferase reporter via Spe *I* and Hind *III* sites. Luciferase reporter assays were performed as following, HEK293 and SW-480 cells were plated in a 96-well plate and co-transfected with 50 nM single-stranded miRNA mimics, or negative control oligonucleotides, with 10 ng of firefly luciferase reporter and 3 ng of pRL-TK (Promega, USA) using the JetPRIME reagent (Polyplus-transfection). Cells were harvested 48 h after the transfection and analyzed using Dual-Luciferase Reporter Assay System (Promega, Japan).

### Oligonucleotide and plasmid transfection

RNA oligos were chemically synthesized and purified by (Genepharma, China). Sense sequence of human miR-320b mimics was 5’- AAA GCU GGG UUG AGA GGG CAA -3’ and antisense sequence was 5’- UUG CCC UCU CAA CCC AGC UUU U-3’. Negative control oligonucleotides were 5’-AAU UCU CCG AAC GUG UCA CTT-3’ and 5’-GUG ACA CGU UCG GAG AAU UTT-3’. The transfections were performed with INTERFERin reagent (Polyplus-transfection). The final concentration of miRNA was found to be 50 nM.

To generate pGL3-c-MYC constructs, the coding DNA sequence fragment of *c-MYC* was amplified and inserted into the *Kpn*I and *Xho*I sites of the pGL3 construct. All constructs were verified by direct sequencing. Finally, the transfections were conducted using INTERFERin reagent (Polyplus-transfection, France). The final concentration of plasmids was diluted to 100 ng.

### MTT assay

To measure *in vitro* growth of CRC cells, the MTT assay was used. A total of 5 × 10^3^ cells were seeded into each well of 96-well plates and transfected with miR-320b mimics or negative control oligonucleotides at a final concentration of 50 nM respectively. On the day of measurement, 100 μl of spent medium was replaced with an equal volume of fresh medium containing 0.5 mg/ml MTT. Plates were incubated at 37 °C for 4 h, then the medium was replaced with 100 μl of DMSO (Sigma, USA), and were then shaken at room temperature for 10 min. Absorbance was then measured at a wavelength of 570 nm.

### Tumorigenicity assay in Non Obese Diabetic (NOD) mice

All mice were cared and maintained according to the National Institute of Health Guide for the Care and Use of Laboratory Animals, with the approval of the Scientific Investigation Board of Second Military Medical University, Shanghai. Cholesterol-conjugated miR-320b mimics and negative control oligonucleotides transfected SW-480 cells (1 × 10^6^) were suspended in 150 μl PBS and then injected subcutaneously into either side of the posterior flank of the same Non obese diabetic (NOD) 6 week old mice. Mice were examined every three days over a course of 4 weeks. Tumor volume (V) was monitored by measuring the length (L) and width (W) of the tumor with calipers and was calculated with the formula *V* = (*L* × *W*2) × 0.5.

### Western blot

Protein from tissue and cells was separated in a 12 % SDS-PAGE gel and transferred onto a nitrocellulose membrane (Bio-Rad, Hercules, USA). The membrane was blocked with 5 % non-fat milk and incubated with anti-c-Myc antibody, anti-Cyclin D1 (Santa Cruz, CA) or anti-β-actin antibody (Sigma, CA, USA). After being washed extensively, a goat anti-mouse secondary antibody (Pierce, IL, USA) was added to the system. The proteins were detected using ECL reagents (Pierce).

### Immunohistochemical (IHC) staining

Paraffin-embedded tissue sections were deparaffinized in xylene and rehydrated in graded series of ethanol followed by heat induced epitope retrieval in citrate buffer (PH 6.0). Sections were incubated at 4 °C overnight with monoclonal antibodies against c-Myc (Santa Cruz, CA) and Ki-67 (Cell Signaling Technology, Danvers, MA). Immunostaining was performed using ChemMate DAKO EnVision Detection Kit, Peroxidase/DAB, Rabbit/Mouse (code K 5007, DakoCytomation, Glostrup, Denmark) according to the manufacturer’s instructions. Subsequently, sections were counterstained with hematoxylin (Dako) and mounted in dimethyl benzene.

### Statistical analysis

All statistical analyses were carried out using the SPSS 16.0 statistical software package. Continuous variables were expressed as mean ± SEM. Differences between groups were calculated with Student’s *t* test. A two-tailed *P* value test was used with a *P* value of < 0.05 considered statistically significant.

## Results

### The expression of miR-320b is down regulated in CRC

**Fig. 1:**
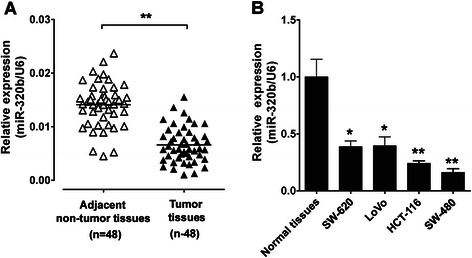
miR-320b is down-regulated in CRC samples and cell lines. **a** Expression of miR-320b was measured in 48 CRC and adjacent non-tumor tissues by qRT-PCR. **b** The expression levels of miR-320b were further measured in 5 normal tissues and 4 CRC cell lines by qRT-PCR. Data analyzed using Student’s *t*-test.*p < 0.05, **p < 0.01

In order to identify the role of miR-320b in CRC carcinogenesis, the expression of miR-320b in CRC samples (patient details have been listed in Table 1) and cell lines was analyzed. As shown in Fig. 1a, miR-320b was significantly decreased in CRC tissues versus adjacent non-tumor tissues. It was also shown that miR-320b was down-regulated in 4 CRC cell lines, when compared with 5 normal colorectal tissues (Fig. 1b). These observations suggest that miR-320b may be an oncosuppressor in CRC.

**Table 1:** Patients’ characteristics of clinical-pathologic features

Characteristics	No. of patients (*n* = 48)	Percent (%)
Age at diagnosis (year)		
≤60	21	43.8
>60	27	56.2
Sex		
Male	25	52.1
Female	23	47.9
Tumor Size(cm)		
≧3 ≤ 3	6	12.5
>3 and ≤5	28	58.3
>5	14	29.2

### miR-320b inhibits CRC growth *in vitro* and *in vivo*

**Fig. 2:**
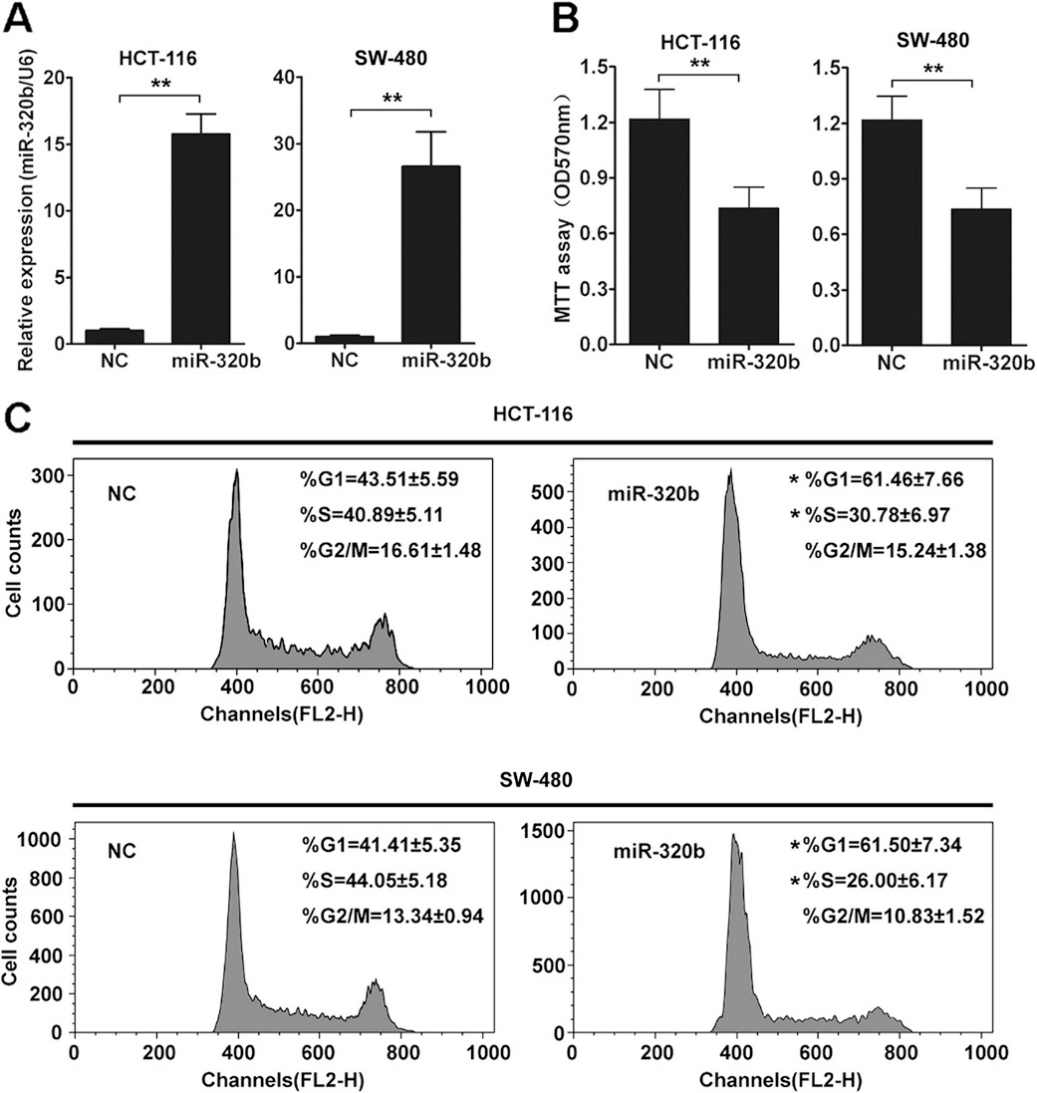
miR-320b overexpression inhibits CRC cells growth *in vitro*. **a** miR-320b expression was quantified by qRT-PCR analysis 48 h. after transfection of miR-320b mimics (miR-320b) and negative control oligonucleotides (NC) in HCT-116 and SW-480 cells. **b** HCT-116 or SW-480 cells were transfected with miR-320b or NC. Cell proliferation was measured using the MTT assay after culturing for 72 h. **c** Cell-cycle distribution was analyzed by FACS analysis. HCT-116 and SW-480 cells were transfected with miR-320b or NC. Nocodazole (25 ng/ml) was added 24 h after transfection for another 16 h, then the supernatant was replaced by fresh medium for 6 h. Data analyzed using Student’s *t*-test.*p < 0.05, **p < 0.01

To investigate the effect of miR-320b on CRC proliferation, miR-320b mimics or negative control oligonucleotides were transiently transfected into two human CRC cell lines, HCT-116 and SW-480. Measurements of RNA expression levels using qRT-PCR (primer sequences have been listed in Table 2) showedd that transfection of miR-320b mimics significantly increased its expression in HCT-116 and SW-480 cells (Fig. 2a). In the cell proliferation assay, restoration of miR-320b in HCT-116 and SW-480 cells resulted in a significant suppression of cell proliferation. The proliferation rate was suppressed in HCT-116 and SW-480 cells after transfection with miR-320b, and the inhibitory efficiencies were 50.0 (*P* < 0.01) and 37.5 % (*P* < 0.01), respectively (Fig. 2b).

**Table 2:** All primers used in this study

Name	Primer sequence
U6 F	5’ - GTGCTCGCTTCGGCAGCACATATAC -3’
U6 R	5’- AAAAATATGGAACGCTTCACGAATTTG -3’
β-actin F	5’-AGAGCTACGAGCTGCCTGAC-3’
β-actin R	5’-AGCACTGTGTTGGCGTACAG-3’
c-Myc (WT) F	5’-GGCTAGTGATGCCCCTCAACGTTAGCTT-3’
c-Myc (WT) R	5’-AAAAGCTT GTAGTCGAGGTCATAGTTCC-3’
c-Myc (MUT) F	5’-GGCTAGTGATCGGGGAGTTGGTTTCGATCACC-3’
c-Myc (MUT) R	5’-AAAAGCTT GTAGTCGAGGTCATAGTTCC-3’
c-Myc (pGL3) F	5’-AAGGTACC ATGCCCCTCAACGTTAGCTTCACC-3’
c-Myc (pGL3) R	5’-AACTCGAGTTACGCACAAGAGTTCCGTAGC-3’
miR-320b F	5’- AAAAGCTGGGTTGAGAGGGCAA-3’

*Abbreviations*: *F* forward primer, *R* reverse primer, *WT* wild type, *MUT* mutant

Inhibition of cell growth in cancer cells is usually associated with concomitant cell cycle arrest. Cell cycle analysis was performed to determine whether the effect of miR-320b on cell proliferation was due to cell cycle arrest. Indeed, the result showed a G1 arrest, with 61.46 % ± 7.66 % of miR-320b-transfected cells in G0/G1 versus 43.51 % ± 5.59 % of control HCT-116 cells. Similar effects of miR-320b were found in SW-480 cells, with 61.5 % ± 7.34 % of miR-320b-transfected cells in G0/G1 versus 41.41 % ± 5.35 % of control cells (Fig. 2c). These results demonstrated that the overexpression of miR-320b inhibits the growth of CRC cells.

**Fig. 3:**
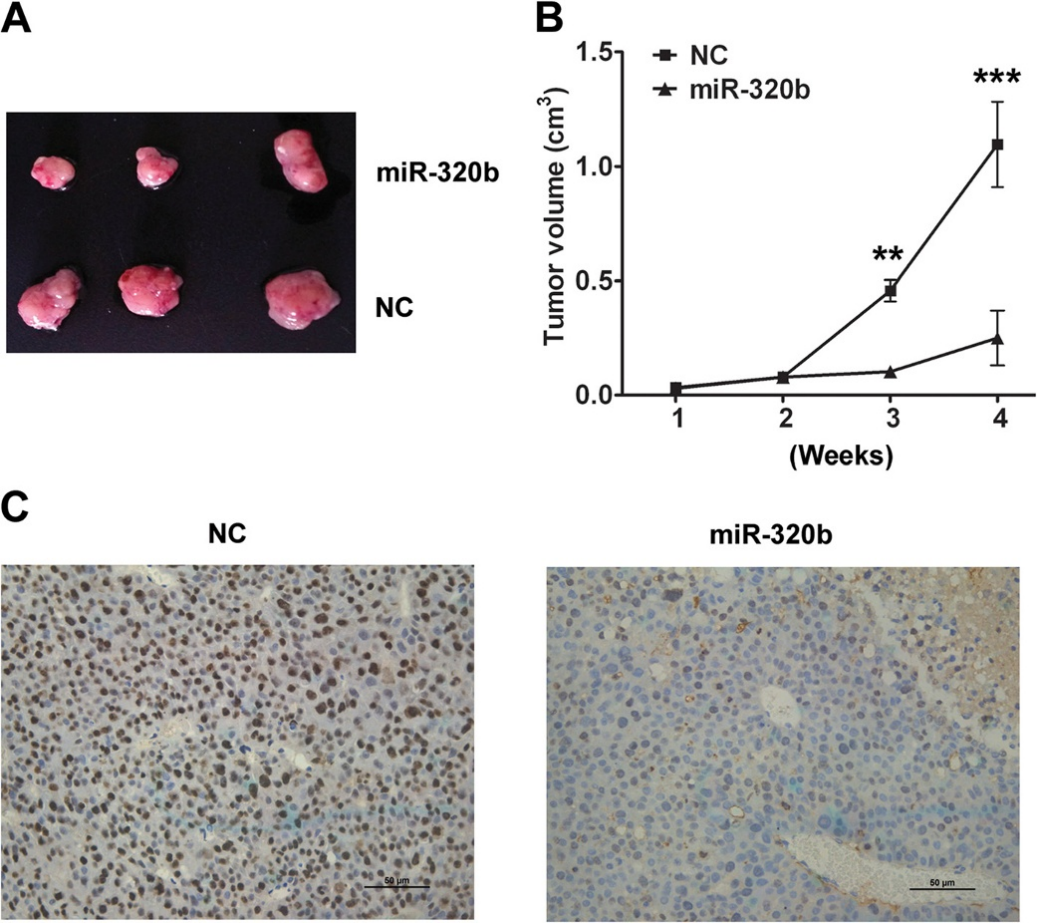
miR-320b overexpression inhibits CRC cells growth *in vivo*. **a** Photographs of dissected tumors from NOD mice 4 weeks after inoculation. **b** The curve of tumor growth. **c** Detection of Ki-67 by IHC in dissected tumor tissues was performed 4 weeks after inoculation. Data analyzed using Student’s *t*-test.*p < 0.05, **p < 0.01

To better understand the modulation of miR-320b in tumorigenisis, we used an *in vivo* model to evaluate the effect of tumorigenicity after overexpression of miR-320b. Cholesterol-conjugated miR-320b mimics and negative control oligonucleotides transfected SW-480 cells were injected subcutaneously into either side of the posterior flank of the same NOD mice. In this tumorigenicity assay (*n* =3), when compared with negative control oligonucleotides (*n* =3) transfectants, miR-320b transfected cells revealed a significant reduction in the tumor size (Fig. 3a, b) and cell proliferation (Fig. 3c), suggesting a potential tumor suppressive effect of miR-320b expression.

### Identification of c-Myc as miR-320b direct target in CRC

**Fig. 4:**
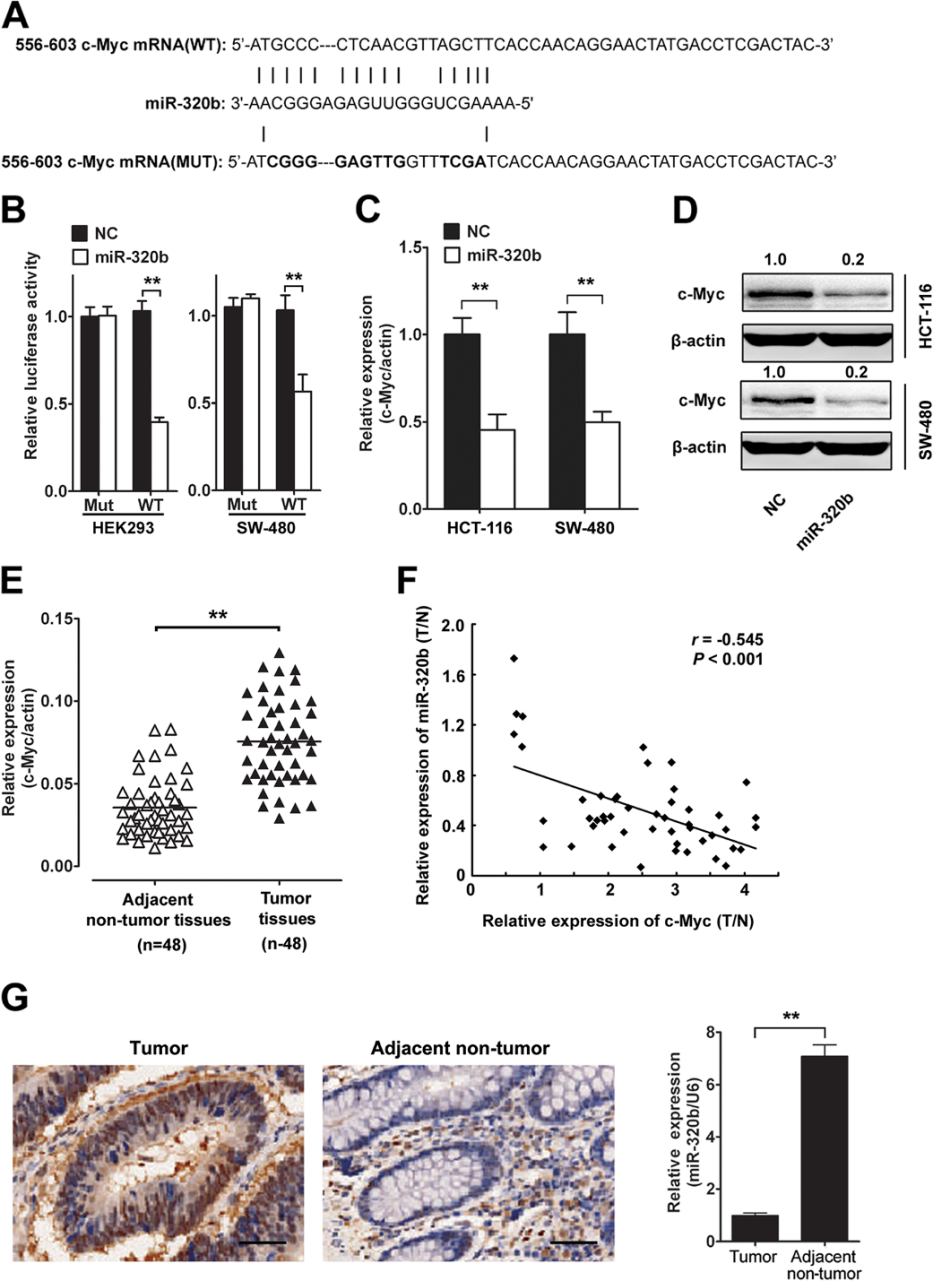
miR-320b down-regulates c-Myc expression in CRC cells. **a** Wild-type (WT) and mutant (Mut) of putative miR-320b targeting sequences in c-Myc mRNA. Mutant sequences were shown in bold type. **b** Analysis of luciferase activity. HEK293 and SW-480 cells were co-transfected with miR-320b mimics (miR-320b) or negative control oligonucleotides (NC), pRL-TK and firefly luciferase reporter plasmid containing putative miR-320b targeting sequences of c-Myc. **c, d** The endogenous c-Myc mRNA **(c)** and protein (**d**) levels were quantified 48 h. after transfection of (miR-320b) and (NC) in HCT-116 and SW-480 cells. **e** Expression of c-Myc was measured in 48 CRC and adjacent non-tumor tissues by qRT-PCR. **f** A negative Spearman correlation between miR-320b and c-Myc mRNA levels was found in CRC samples. **g** Analysis of c-Myc and miR-320b expression in the same CRC and adjacent non-tumor tissue by IHC and qRT-PCR. Brown signal in IHC was considered as positive staining for c-Myc. Scale bar = 50 μm. Data analyzed using Student’s *t*-test.*p < 0.05, **p < 0.01

To confirm miR-320b regulation of the expression of *c-MYC* gene, we first performed luciferase reporter assays in HEK293 and SW-480 cells. As shown in Fig. 4a and b, transfection of miR-320b caused a significant decrease in luciferase activity in cells transfected with the reporter plasmid with wild type targeting sequence of *c-MYC* mRNA but not reporter plasmid with mutant sequence of *c-MYC*.

HCT-116 and SW-480 cells which demonstrated a lower expression of miR-320b (Fig. 1b) were transfected with miR-320b mimics or negative control oligonucleotides, and *c-MYC* mRNA as well as protein levels were examined by qRT-PCR and confirmed via western blotting. The levels of *c-MYC* mRNA and protein were consistently and substantially down regulated by miR-320b (Fig. 4c, d). Moreover, *c-MYC* mRNA levels were significantly increased in CRC tissues versus adjacent normal tissues which agreed with previous report [16]. Consistent with this finding, Fig. 4f showed a reversed correlation between miR-320b expression levels and *c-MYC* mRNA levels in CRC tissues(*r* = −0.545, *P* < 0.001). CRC tissues with low miR-320b consistently showed much higher *c-MYC* expression compared to normal tissues with high levels of miR-320b, but lower *c-MYC* expression (Fig. 4g). Taken together, our results demonstrated that c-Myc was a direct target of miR-320b in CRC cells.

### Restoration of miR-320b suppresses c-Myc-induced CRC proliferation

**Fig. 5:**
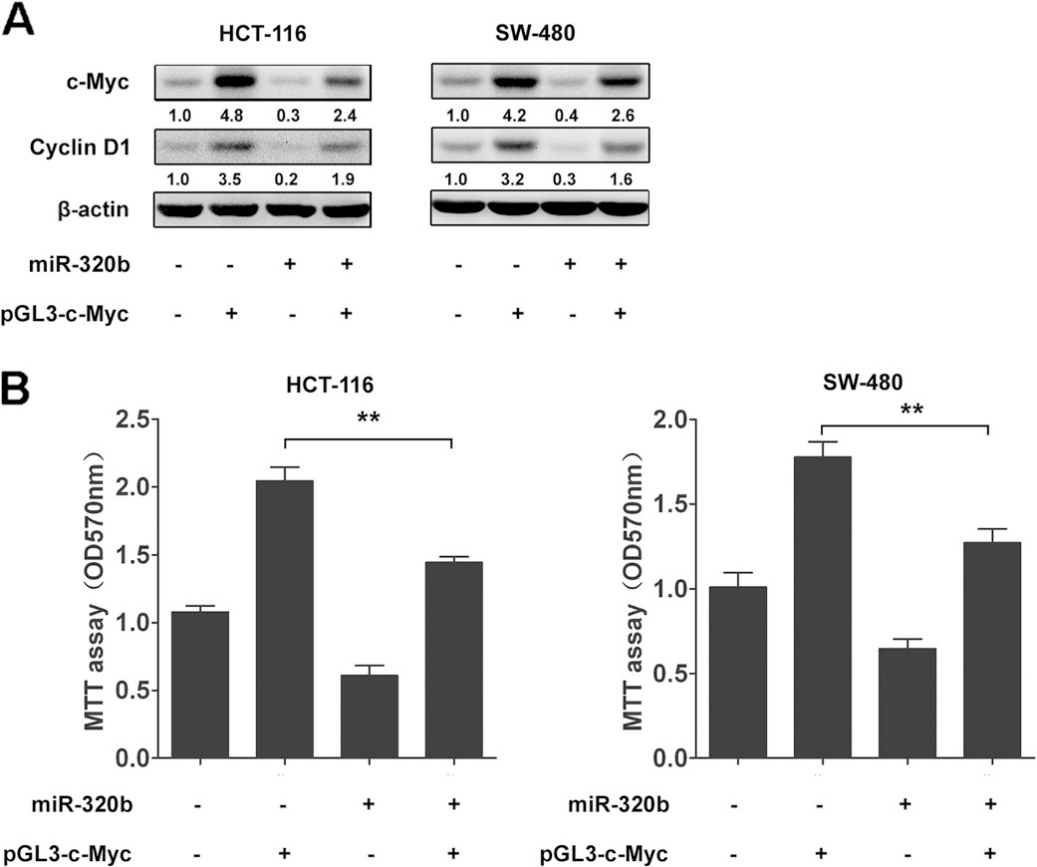
miR-320b inhibits the proliferation of CRC cells by targeting c-Myc. **a** HCT-116 and SW-480 cells were transfected with miR-320b and/or pGL3-c-Myc, 48 h. later the expressions of c-Myc and Cyclin D1 were analyzed via western blotting. **b** HCT-116 andSW-480 cells were transfected with miR-320b and/or pGL3-c-Myc, and cell proliferation was measured by by MTT assay at 72 h. after transfection. Data analyzed using Student’s *t*-test.*p < 0.05, **p < 0.01

This study has shown that overexpression of miR-320b suppressed the proliferation of CRC cells, and c-Myc was a direct target of miR-320b. We next explored the inhibitory effect of miR-320b on CRC cells’ viability. It was found that c-Myc protein levels in HCT-116 and SW-480 cells significantly increased as well as its targeting genes such as Cyclin D1(key factor in cell growth regulation [17, 18]). At the mean time, restoration of the miR-320b expression levels in HCT-116 and SW-480 cells showed a markedly inhibited expression of c-Myc and Cyclin D1 (Fig. 5a). In line with these findings, miR-320b significantly suppressed c-Myc-induced cell proliferation in HCT-116 and SW-480 cells (Fig. 5b). Together, these results demonstrated that miR-320b could regulate CRC cells growth through targeting c-Myc.

## Discussion

In this study, we examined the expression profile of miR-320b in CRC. Our results showed that miR-320b was down-regulated in CRC tissues as well as in 2 human CRC cell lines, which was similar with previous reports [12]. In addition, we identified c-Myc as a direct and functional target of miR-320b in CRC cells. Overexpression of miR-320b in CRC cells significantly inhibited the cell proliferation by down-regulating c-Myc.

The interest in understanding the role of miRNA in cancer cells and carcinogenesis has grown rapidly, so has the amount of studies in this field [9, 10]. There has been reports showing that miR-320 is down regulated in several tumor types [12–15], and one of miR-320 ‘s downstream effects was inhibiting cell proliferation by targeting transferrin receptor 1 (CD71) in human leukemia cell line HL-60 [19]. Additionally, it is known that miR-320 suppresses the stem cell like characteristics of prostate cancer cells by down regulating the Wnt/beta-catenin signaling pathway [20].

Deregulation of miRNAs has been indicated to be heavily involved in tumorigenesis and tumor development [10, 21, 22]. Our results showed that miR-320b was down-regulated in CRC, which prompted us to further investigate the role of miR-320b in CRC cells. In order to explore the underlying mechanisms of the silenced miR-320b in CRC, the CRC pathogenesis-related target genes were further analyzed. In a recent study, 18,000 high-confidence miRNA-mRNA interactions in HEK 293 cells by CLASH were reported [23]. However, it remains unclear whether these miRNA pairings also exist in CRC cells. The CLASH data showed that 72 genes were targeted by miR-320b. Among these genes, we focused on *c-MYC*, a central component of the MAPK/ERK pathway, which is involved in many cellular processes including cell growth, cell differentiation, apoptosis and other cellular functions [17]. From the CLASH data in HEK293 cells, the potential targeting sequence for miR-320b with a calculated energy of −23.1 kcal/mol is within the protein coding region of c-Myc mRNA from 556 to 603 [23]. This targeting mode is similar to miR-185-3p (a potential target sequence in the protein coding region of c-Myc mRNA [24]), while distinct from other c-Myc targeting miRNAs such as miR-145, let-7a, miR-24(target sites in 3’UTR of c-Myc [25–27]). However, the role of miR-320b in CRC is not fully understood because of the lack of target gene information.

Our results demonstrated that c-Myc was directly targeted by miR-320b in CRC cells. Overexpression of miR-320b in CRC cells decreased both mRNA and protein levels of *c-MYC*, a finding that is consistent with a previous work reporting a negative correlation of miR-320b with various genes including *c-MYC* in CRC [28]. Our results suggest that miR-320b is a candidate tumor suppressor in the pathogenesis of CRC.

*c-MYC* is a node gene in multiple pathways, and most of its target genes including *CCND1* are crucial for cell growth, proliferation and development [29–31]. As a regulator gene, *c-MYC* is carefully managed at different levels [32]. Recent studies have shown that the deregulation of miRNAs is responsible for high levels of c-Myc in many cancers. Several miRNA are known to be regulators of c-Myc in various different cancers including miR-24 in leukemia [26], miR-145 in oral squamous cell carcinoma [25], let-7a in burkitt lymphoma [27], miR-34a in renal cell carcinoma [33] and miR-185-3p in CRC [24]. In this study, c-Myc was identified to be directly targeted, and regulated by miR-320b in CRC cells. Our results agreed with the viewpoint that a single mRNA molecule can been regulated by multiple miRNA genes in different cells [34, 35].

## Conclusions

We conclude that miR-320b is frequently down regulated in CRC cell lines and clinical samples. Our data demonstrated that c-Myc was directly targeted and regulated by miR-320b, and that overexpression of miR-320b significantly inhibited CRC cells proliferation through regulating c-Myc. Our identification of c-Myc as a target of miR-320b provides new insights into the pathophysiology of CRC proliferation and identifies miR-320b as a novel therapeutic targets for the treatment of CRC.

## Abbreviation

CRC: Colorectal cancer
mRNA: Messenger RNA
qRT-PCR: Quantitative real-time PCR
miR-320b: MicroRNA-320b
IHC: Immunohistochemistry
CLASH: Crosslinking, ligation, and sequencing of hybrids
